# Defining the timeline of periostin upregulation in cardiac fibrosis following acute myocardial infarction in mice

**DOI:** 10.1038/s41598-022-26035-y

**Published:** 2022-12-18

**Authors:** Hadas Gil, Matan Goldshtein, Sharon Etzion, Sigal Elyagon, Uzi Hadad, Yoram Etzion, Smadar Cohen

**Affiliations:** 1 Avram and Stella Goldstein-Goren Department of Biotechnology Engineering, Ben-Gurion University of the Negev, POB 653, 8410501 Beer-Sheva, Israel; 2 Regenerative Medicine and Stem Cell (RMSC) Research Center, Ben-Gurion University of the Negev, 8410501 Beer-Sheva, Israel; 3 Department of Physiology and Cell Biology, Ben-Gurion University of the Negev, 8410501 Beer-Sheva, Israel; 4 The Ilse Katz Institute for Nanoscale Science and Technology, Ben-Gurion University of the Negev, 8410501 Beer-Sheva, Israel

**Keywords:** RNA, Myocardial infarction, Myocardial infarction, Gene expression, Reverse transcription polymerase chain reaction, Biomarkers, Target identification, Fluorescence imaging

## Abstract

After myocardial infarction (MI), the heart's reparative response to the ischemic insult and the related loss of cardiomyocytes involves cardiac fibrosis, in which the damaged tissue is replaced with a fibrous scar. Although the scar is essential to prevent ventricular wall rupture in the infarction zone, it expands over time to remote, non-infarct areas, significantly increasing the extent of fibrosis and markedly altering cardiac structure. Cardiac function in this scenario deteriorates, thereby increasing the probability of heart failure and the risk of death. Recent works have suggested that the matricellular protein periostin, known to be involved in fibrosis, is a candidate therapeutic target for the regulation of MI-induced fibrosis and remodeling. Different strategies for the genetic manipulation of periostin have been proposed previously, yet those works did not properly address the time dependency between periostin activity and cardiac fibrosis. Our study aimed to fill that gap in knowledge and fully elucidate the explicit timing of cellular periostin upregulation in the infarcted heart to enable the safer and more effective post-MI targeting of periostin-producing cells. Surgical MI was performed in C57BL/6J and BALB/c mice by ligation of the left anterior descending coronary artery. Flow cytometry analyses of cells derived from the infarcted hearts and quantitative real-time PCR of the total cellular RNA revealed that periostin expression increased during days 2–7 and peaked on day 7 post-infarct, regardless of mouse strain. The established timeline for cellular periostin expression in the post-MI heart is a significant milestone toward the development of optimal periostin-targeted gene therapy.

## Introduction

Heart failure is a clinical syndrome that affects over 25 million people worldwide^1,2^. Typically caused by myocardial infarction (MI) due to an occlusion of coronary arteries, heart failure results in the extensive hypoxia-induced death of the cardiomyocytes that populate the infarction area^3^. Because of the adult heart's limited regenerative capacity^4^, cells that were lost to MI are replaced during the wound healing process by resident fibroblasts. Throughout this process, macrophages initiate wound repair by clearing debris and releasing growth factors (GFs). Influenced by the indirect paracrine signal of the secreted GFs, fibroblasts are recruited to the injury site, and activated to proliferate and secrete type I and type III collagen to the extracellular matrix (ECM). Activated fibroblasts then undergo conversion to myofibroblasts expressing alpha smooth muscle actin (αSMA)^5^, and collagen deposition continues, creating a fibrous scar known as fibrosis^6,7^. The so-called replacement fibrosis process, i.e., scar formation, is essential to prevent ventricular wall aneurysm and rupture after ischemic insult^8^. Nevertheless, the increase in mechanical stress due to wall thinning in combination with specific hormonal and paracrine mediators induces further expansion of fibrosis to remote heart regions. This reactive fibrosis process that occurs in the infarct border zone and in the remote, uninjured myocardium is responsible for the remodeling of the tissue. The remodeled tissue, however, exhibits altered chamber compliance and increased ventricular stiffness, which compromise cardiac output and eventually lead to heart failure and an increased risk of death^7,9^.

Studies have demonstrated that periostin, a 90-kDa non-structural matricellular protein^10,11^, is a major effector in MI-induced fibrosis^12–14^. While periostin is absent in the healthy myocardium, pathological insult such as MI stimulates periostin production by the activated fibroblasts, which secrete it to the ECM^15^. Periostin was found to be positively correlated with increasing levels of collagen^13,14^, and upregulated in mouse models of fibrosis-associated-hypertrophic cardiomyopathy^2^. Furthermore, periostin interaction with fibrillar collagens, either directly or with the aid of specialized proteins, mediates collagen cross-linking in the ECM. This not only promotes the deposition and stabilization of the scar, but also ensures the persistent presence of periostin in the tissue^16^. For these reasons, periostin has been marked as a candidate therapeutic agent for the regulation of fibrosis to tackle the related problem of adverse remodeling in the diseased heart^17,18^.

Despite the potential health benefits of post-MI periostin targeting, the technique has not been properly adapted to the relevant stage of wound healing. Although complete periostin removal in mice was reported to decrease MI-induced fibrosis and to improve ventricular function, it was also associated with an increased incidence of wall rupture^2,19^, jeopardizing their survival during the early stages of healing (first 10 days post-MI). These two confounding conditions, both of which entail periostin expression, contrast periostin's positive effect in the short term post-MI (promotes wound healing) with its negative effect over the long term (stimulates reactive fibrosis)^12^. Alternately, selective ablation of *Postn*-expressing cardiac fibroblasts (CFs) executed on days 7–14 after the MI overlooked the possibility that the reactive fibrosis may occur earlier^20^, as fibroblast activation was shown to begin on day 2, after which the fibroblasts transitioned to myofibroblasts on day 4^21^. To minimize the extent of remodeling without impairing scar stability, periostin regulation must be precisely timed.

In this study, therefore, we aimed to elucidate the timeline of periostin expression after MI to maximize the treatment effects of time-based periostin manipulation. The present paper describes the limited period after MI during which periostin is produced by focusing on the levels of the periostin gene and its protein in the *cell*, where periostin is originally produced, rather than quantifying its levels in tissues^22^, which could result in an inaccurate estimate. In this study, C57BL6J and BALB/c mice were subjected to surgical MI via left coronary artery ligation. Thereafter, periostin was quantified at selected time points, first by expression of mRNA extracted from the tissue^23^ (including all cell types), and later by cellular protein expression, particularly in cardiac fibroblasts. The results show increased periostin gene and protein expression during days 2–7 post-MI, and its maximum upregulation was measured on day 7 in all of the mouse strains. On day 14, periostin production was relatively lower. The established timeline for cellular periostin expression in the post-MI heart facilitates the design of a better periostin-targeted gene therapy that will correspond to the optimal timeframe for intervention. This may ameliorate cardiac repair without hampering wall stability.

## Materials and methods

For additional details about the methods, refer to the Supplementary Information.

### Animals

C57BL/6J and BALB/c female mice (11–12 weeks old, weight 22–30 g) were purchased from Envigo (Jerusalem, Israel). All experiments were performed with the approval and according to the regulations of the Institutional Animal Care and Use Committee of Ben-Gurion University of the Negev, as well as in accordance to the ARRIVE guidelines. The study was executed on two mice strains: C57BL/6J and BALB/c, commonly used in myocardial infarction studies. While C57BL/6 is often utilized for genetically manipulated models, BALB/c females were shown to be preferable for studies of the later stages of MI due to their lower rates of infarct rupture and more apparent cardiac remodeling compared with males and other mice strains^24^.

### Experimental myocardial infarction (MI)

MI was induced by permanent ligation of the left anterior descending artery (LAD). A mixture of isoflurane (3–5%) in O_2_ was used to anesthetize mice, which were intubated and ventilated with the isoflurane (2–2.5% after mixing with O_2_) by using a rodent ventilator as previously described^23^. Mice chests were surgically opened by left thoracotomy through the fourth intercostal space, the pericardium was removed, and the left main coronary artery was permanently occluded by an intramural suture (8–0 polypropylene). Successful artery occlusion was visualized by immediate discoloration of the myocardium, and further validated by echocardiography 24–72 h post-occlusion. For echocardiography, anesthesia was first induced with 3% isoflurane mixed with 0.5 L/min 100% O_2_, after which the isoflurane concentration was reduced to 1.5% to maintain a steady-state sedation level. Echocardiograms were performed with a commercially available echocardiography system (Vevo 3100, VisualSonics, Toronto, ON, Canada). The heart was first imaged in the 2D mode in the parasternal long and short axis views of the LV. M-mode images were obtained at the level of the papillary muscles. Care was taken to avoid excessive pressure on the mice during the test. LV TRACE analysis was then performed on parasternal long axis images. LV TRACE Left Ventricular Ejection Fraction (EF), indicating the percentage of blood leaving the heart in each contraction, was automatically calculated by the system as EF% = [(LVEDV − LVESV)/LVEDV] × 100, wherein LVEDV stands for LV end diastolic volume and LVESV is LV end systolic volume. EF measurements were averaged for three consecutive cardiac cycles. C57BL/6J mice that exhibited visualized contraction damage and BALB/c mice whose EF values were below 40% were approved for the study. For additional details about the methods, refer to the Supplementary Information.

### Tissue processing and laser capture microdissection (LCM)

C57BL/6J female mice were sacrificed under deep anesthesia at several different points in time after the MI. As previously described^23^, the removed hearts were cut laterally into two pieces (apex and middle section), washed immediately in ice cold phosphate-buffered-saline-diethylpyrocarbonate (PBS-DEPC, BI, Kibbutz Beit-Haemek, Israel) and embedded in optimum cutting temperature compound (OCT, Sakura (Alphenaan den Rijn)). Samples were snap-frozen in liquid N_2_ and stored at − 80 °C until sectioning. The frozen mouse hearts were cut into 10-μm-thick sections using a Leica 3500S cryostat (Leica, Germany). Sections were placed on pre-treated membrane slides from PALM Technologies (Bernreid, Germany) and processed within 24 h. Frozen sections were stained for nuclei using 19 cresyl violet, fixed with ice-cold 70% ethanol for 2–3 min and dipped in ice-cold DEPC RNase-free water to remove OCT. The slides were then dipped in cresyl violet for 15 s; excess stain was removed on an absorbent surface and washed by dipping in 70% and 100% ethanol. Samples were then air-dried and LCM was performed using the PALM laser microdissection system (Zeiss). Cut elements were catapulted into adhesive caps situated directly above the section. The area of injury in the left ventricle (MI zone) was that with the higher nuclear density (due to infiltrating inflammatory cells) on days 4–7, while on days 14–28, it was defined by the borders of the scarred region.

### Quantitative reverse transcriptase polymerase chain reaction analysis

Total RNA in the LCM samples was isolated by using the miRNeasy Micro Kit (Qiagen). Reverse transcription (cDNA synthesis) was performed using a high-capacity cDNA reverse transcription (RT) kit (Applied Biosystems, Foster city, CA) according to the manufacturer's protocol with an initial amount of RNA of 100 ng (measured by NanoDrop™). qPCR was performed using Taqman gene expression assays (Thermo-fisher, Table S1), and the qPCR results were normalized using a housekeeping gene (endogenous control: HPRT). For gene expression analysis, mRNA levels were determined by real-time PCR using StepOnePlus™ Applied detection system according to the manufacturer's instructions for comparative ΔΔCT (Applied Biosystems). Each 10-μl reaction contained 2 μl cDNA (5 ng). For additional details about the methods, refer to the Supplementary Information.

### Cell extraction and flow cytometry

C57BL/6J and BALB/c female mice were sacrificed using isoflurane at different time points following the MI. CFs were isolated from the extracted hearts by the following process: First, the hearts were washed in cold PBS while applying successive pressings to remove the excessive blood. Aorta and atriums were removed, and each heart was minced to ~2 mm pieces and suspended in 3 ml enzyme solution of 1 mg/ml Liberase^®^ Thermolysin High (Sigma-Aldrich-Merck, Rehovot, Israel) and 10 μM CaCl_2_ in HBSS, inserted with DNAse I (40 µg/ml, StemCell Technologies) to reduce aggregates. The entire content was pipetted 12 times using a 5 ml pipette, and put in an orbital incubator shaker for 15 min (85 RPM, 37 °C). Pipetting and shaking were repeated twice more such that the total total shaking time was 45 min. Next, the solution was pipetted an additional 30 times by using a 1000-μl pipette. The digested tissue was filtered through a 70-µm cell strainer followed by low-speed centrifugation (50 g, 2 min, RT) to expel the precipitated cardiomyocytes, cell clumps and remaining undigested tissue. After a second filtration step through a 40-μm filter (450 g, 4 min, 4 °C), the cell pellet was washed with PBS and suspended in EDTA-flow-cytometry buffer (PBS (BI) supplemented with 2% fetal bovine serum (FBS, BI) and 1 mM EDTA (Sigma-Aldrich-Merck)). From this moment and throughout the entire duration of sample preparation for flow cytometry, the freshly isolated cells were kept on ice and protected from light. To prevent the nonspecific binding of the FC antibody region to an FC receptor, cells were incubated with FC blocker solution [anti-mouse CD16/CD32 (1:50; Clone 93, Biolegend) diluted in flow-cytometry buffer (PBS supplemented with 2% FBS)] for 5 min. The LIVE/DEAD™ Fixable Violet Dead Cell Stain Kit (Thermofisher Scientific) was then used to distinguish live/dead cells. To stain the MEFSK4 surface markers, cells were incubated for 30 min with APC-conjugated-MEFSK4 rat antibody (1:10, 130-102-900, Miltenyi Biotec, Bergisch Gladbach, Germany). APC-conjugated-normal IgG1 rat antibody (1:10, 130-102-646, Miltenyi Biotec) was used as the isotype control. Next, cells were fixed in 3.7% formaldehyde (Sigma-Aldrich-Merck) for 15 min, permeabilized with BD Perm/Wash™ Buffer (1:10, 554714, BD Biosciences, San Jose, CA), and, to stain the periostin, incubated for 30 min with Alexa Fluor^®^ 488 conjugated periostin mouse antibody (1:50, sc-398631, Santa Cruz biotechnology, Dallas, Texas). Alexa Fluor^®^ 488 conjugated normal IgG1 mouse antibody (1:50, sc-3890, Santa Cruz) was used as the isotype control. Resuspended in flow cytometry buffer, cell samples were analyzed with BD FACSAria™ III and BD FACSDiva software version 8.0.1 (BD Biosciences). FlowJo (version 10) was used for data analyses: live cells that exhibited higher fluorescence levels compared with the isotype control of one of the tested markers were considered to be positive for that marker. To verify the credibility of cell identification, unstained cells were also analyzed via ImageStreamX Mark II (Amnis, Seattle, WA). Cell acquisition and analysis were performed using the INSIPE Application, version 6.0.

### Statistical analysis

Statistical analysis was performed with GraphPad Prism version 6.01 for Windows (GraphPad Software, San Diego, CA). Quantitative gene expression results were compared by one-way ANOVA with Kruskal–Wallis's multiple comparisons test and were displayed as the means ± standard deviation (SD) of multiple (n ≥ 3) independent experiments. Histograms depicting Ex vivo post-MI data are presented as means ± SD of n = 3 mice from each group. Data were considered statistically significant when P < 0.05.

## Results

Periostin expression by activated CFs was first observed in cell cultures during preliminary experiments (Supplementary Information). Primary CFs from healthy C57BL/6J male mice were stimulated for 24–48 h with TGF-β1, which is associated with post-MI cardiac fibrosis^3,6,8^. The results showed increased expression of the periostin and α-SMA genes (Supplementary Figs. S1–S2). The correlation between TGF-β1-stimulation and the expression of these fibrotic genes signals that they are representative indicators for the activated profile of fibroblasts in the ischemic heart^3,6,8^. The observation that in our results, periostin was the most significantly upregulated gene motivated us to explore its relevance in cardiac fibrosis through time after MI.

### Timeline of periostin upregulation in infarcted areas identified by gene analysis

**Figure 1:**
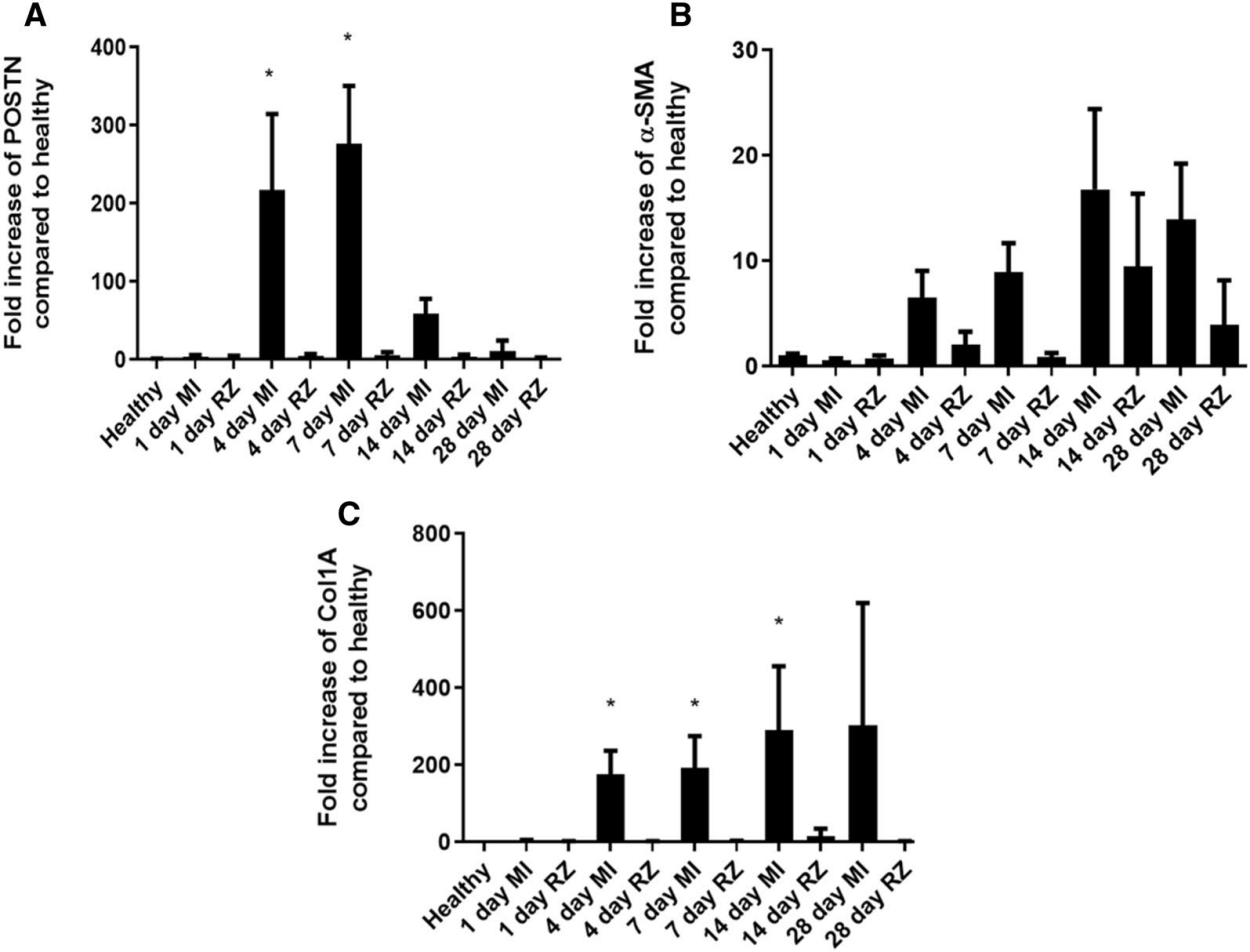
Periostin and fibrotic gene expression timelines after MI at infarct (MI) and remote zone (RZ). Cardiac tissue sections collected with the LCM system from MI-induced C57BL/6J female mice were analyzed for (**A**) periostin, (**B**) α-SMA and (**C**) Collagen type IA by qRT-PCR. Levels were normalized to healthy hearts (without MI). Gene levels, which were normalized to the HPRT housekeeping gene, are represented relative to the gene level of healthy hearts. Data are expressed as the means ± SD (n = 3) *p < 0.05, (Kruskal–Wallis’s multiple comparisons test relative to healthy controls, one-way ANOVA).

As a first step to elucidate the exact timing of periostin cellular expression after MI, we conducted qRT-PCR on cells procured from the infarcted cardiac tissue of C57BL/6J female mice (Fig. 1). Using the LCM system as previously described^23^, we separately collected two tissue sections—the injured (infarcted) area and the remote area—from the myocardial wall of the left ventricle at several time points after the MI. RNA was extracted and the gene expression timeline of periostin (and of the fibrotic genes Col1a and α-SMA, based on our preliminary results in TGF-β1-activated fibroblasts) was evaluated by qRT-PCR. In the infarcted zone (MI), periostin gene expression level was strongly elevated on day 4 after MI (more than a 200-fold increase compared to healthy myocardium), and it continued to rise until day 7, after which it decreased (day 14). By day 28, periostin's gene expression level returned to its non-ischemic profile, while in the remote myocardium (RZ), it exhibited negligible expression levels in all of the time periods (Fig. 1A). Col1a and α-SMA gene expression levels showed a more persistent incline with time that was maintained until day 28, most prominently in the MI zone (Fig. 1B,C) due to the activated cell phenotype in this area.

### Extraction and identification of fibroblasts from the mouse heart

**Figure 2:**
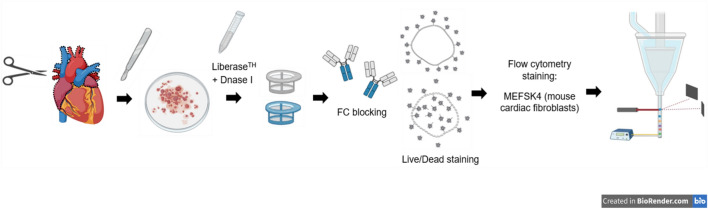
Schematic protocol for the isolation and identification of cardiac fibroblasts: 11–12 week-old C57BL/6J and BALB/c female wild-type (WT) mice were sacrificed, and the following process was executed separately on each mouse: the heart was extracted and washed with PBS. Aorta and atriums were removed, and the ventricles were then minced and enzymatically digested using HBSS complemented with a mixture of collagenase I, II, and DNAse, followed by 70 µm and 40 µm filtrations and FC blocking. Live/dead staining and staining with the antibody MEFSK4 that is specific for CFs enabled us to examine them using flow cytometry. Figure was created using BioRender.com.

**Figure 3:**
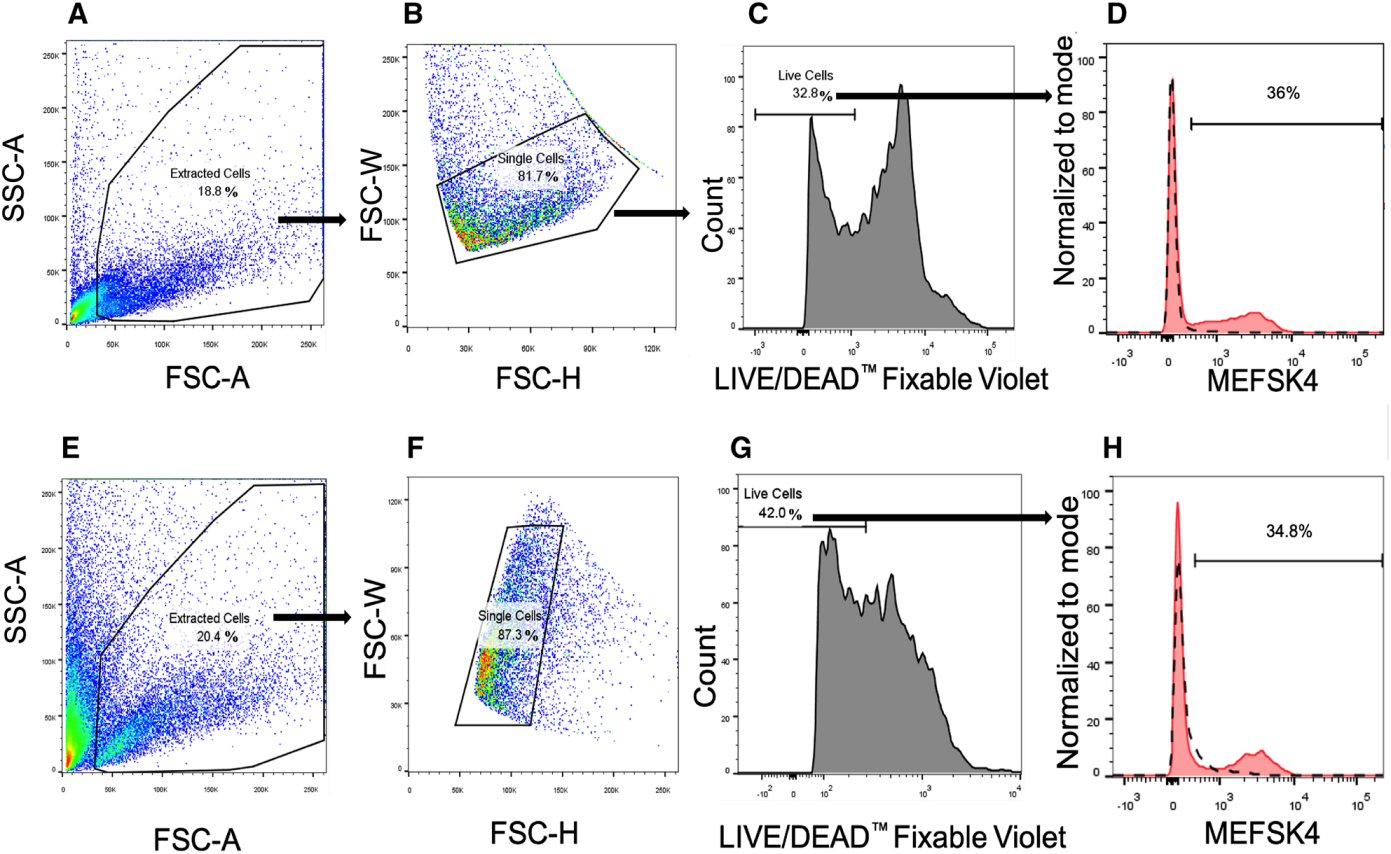
Cardiac MEFSK4 + represents the fibroblast subpopulation in the mouse heart that was portrayed by flow cytometry analyses. (**A–D**) Representative plots and quantification of flow cytometry analysis of healthy hearts from C57BL/6J female mice (*n* = *3)*: (**A**) cellular debris below 50 k in FSC-A were excluded in FSC-A versus SSC-A plots, and (**B**) cell clusters were dismissed in FSC-H versus FSC-W plots; (**C**) live cells were selected by weaker LIVE/DEAD™ Fixable Violet stain fluorescence according to a corresponding histogram; finally, (**D**) the percentage of MEFSK4+ cells was quantified from the population of live cells, with cardiac MEFSK4+ representing the fibroblast subpopulation among the non-myocyte cells in the mouse heart. (**E–H**) Representative plots and quantification of flow cytometry analysis of healthy hearts from BALB/c mice (*n* = *3)*: (**E**) cellular debris below 50k in FSC-A were excluded in FSC-A versus SSC-A plots, and (**F**) cell clusters were dismissed in FSC-H versus FSC-W plots; (**G**) live cells were selected by weaker LIVE/DEAD™ Fixable Violet stain fluorescence according to a corresponding histogram; finally, (**H**) the percentage of live cells that comprised cardiac MEFSK4+ (%) cells was quantified; cardiac MEFSK4+ cells constitute the fibroblast subpopulation of the non-myocyte cells in the mouse heart.

**Figure 4:**
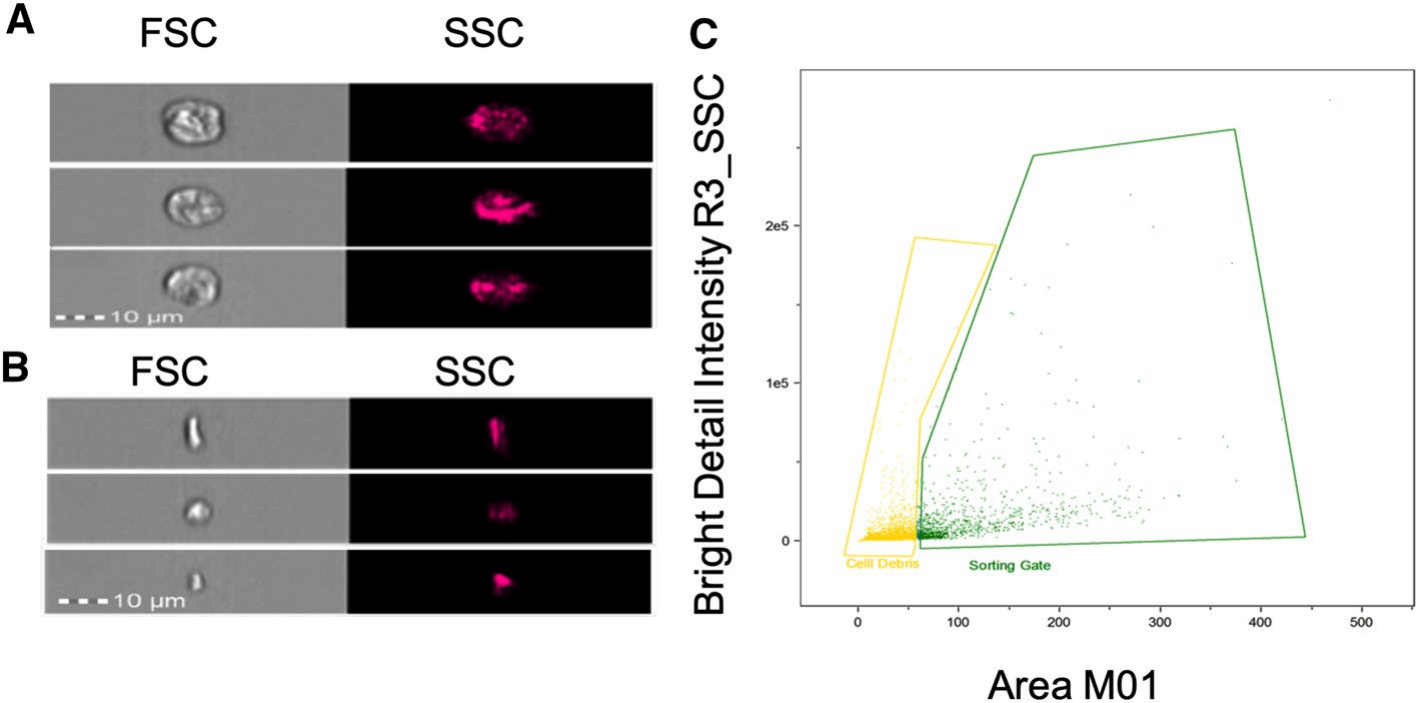
Cellular debris exclusion from freshly extracted non-myocyte cardiac cells as illustrated by ImagestreamX. Cells were extracted from BALB/c female mice (*n* = *3)*: (**A)** brightfield (BF) and darkfield (SSC) images of representative cells. (**B**) Brightfield (BF) and darkfield (SSC) images of representative cellular debris. (**C**) Area of the brightfield image plotted versus bright detail intensity (BDI). The percentage of cells from the extraction product is shown in green (%), and the percentage of cellular debris is shown in yellow (%).

To examine whether periostin upregulation in the adult ischemic heart is produced mainly by activated fibroblasts^5,12,15,25,26^, we aimed to reinforce the described timeline of post-MI periostin expression specifically in CFs. To this end, we focused on obtaining a cell composite, which would be suitable for flow cytometry analysis, enabling both the identification of fibroblasts and the quantification of periostin expression levels among them. The extraction of CFs from the whole hearts of wild-type female mice (C57BL/6J and BALB/c) was therefore optimized (Fig. 2): digested cell suspension was first gated in FSC-A, SSC-A plots to exclude cellular debris (Fig. 3A,E). Cellular debris was classified below 50k in FSC-A, and to avoid substantial cell loss and verify that only debris is omitted under this strategy, the primary cell suspension was also viewed with an imaging flow cytometer (Fig. 4). Brightfield (transmission) and darkfield (side-scatter) images allowed us to qualitatively distinguish isolated cells and cellular debris, respectively, by size, morphology and granularity (Fig. 4A,B). Recorded and focused events were all plotted together by area of the brightfield image (corresponding to FSC-A) versus bright detail intensity (corresponding to SSC-A), and the described gating strategy was applied (cellular debris was classified below 50k in the area axis). Slightly more than 90% of gated debris was in fact recognized as cellular debris (data not shown), thereby demonstrating the feasibility of debris exclusion by the proposed method. Following debris elimination, single cells were gated in FSC-H, FSC-W plots to dismiss cell clusters (Fig. 3B,F). Subsequently, live cells were recognized by dim LIVE/DEAD™ Fixable signal fluorescence (Fig. 3C,G). Lastly, to identify the fibroblast subpopulation, live cells were surface stained for MEFSK4, a known marker for murine cardiac resident fibroblasts, independent of their activation status^27–29^ (Fig. 3D,H). In both female mouse strains, ~35% of the live cells were positive for MEFSK4, and therefore, they were categorized as fibroblasts.

### Periostin upregulation timeline in CFs is identified by flow cytometry analysis

**Figure 5:**
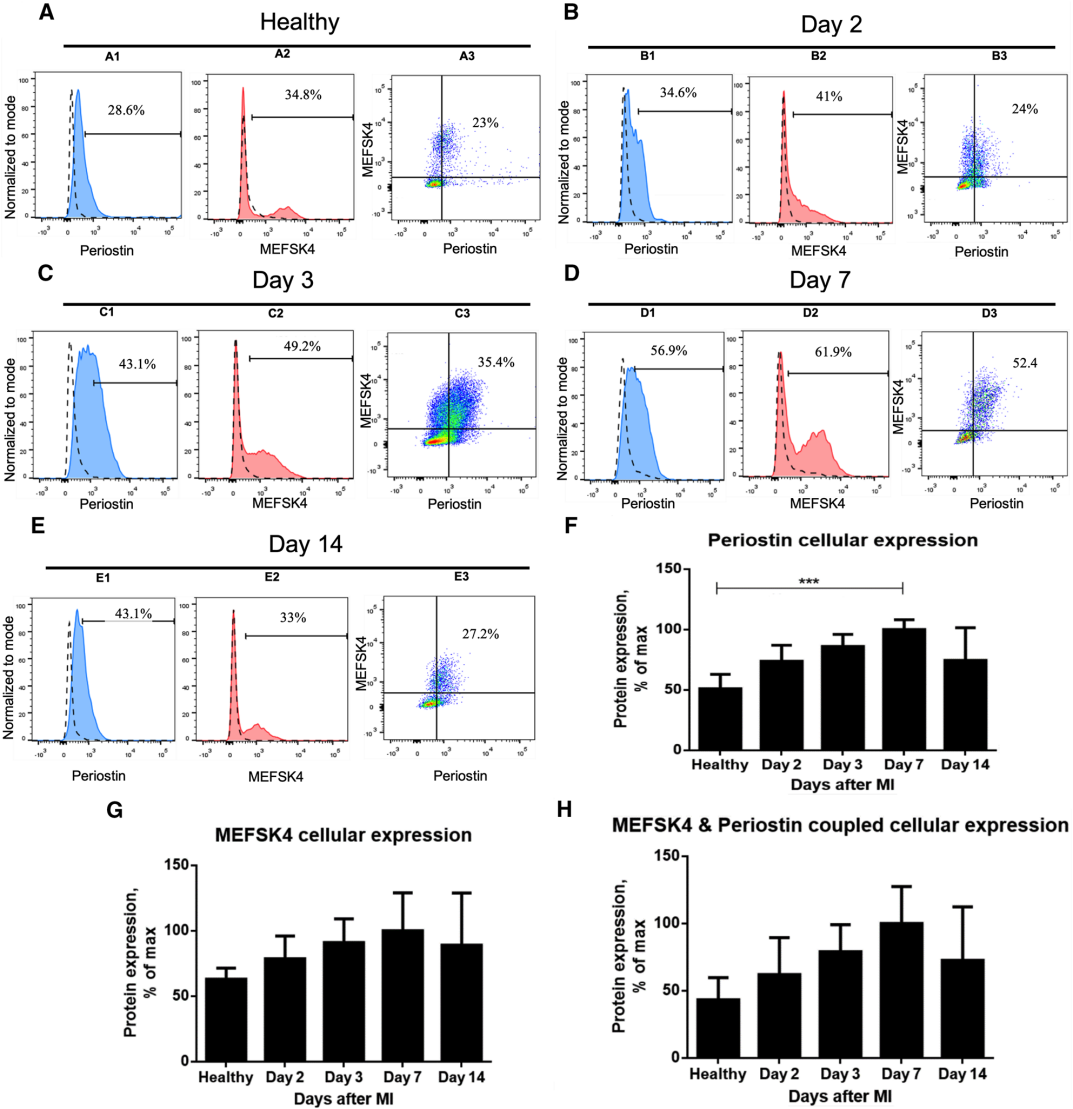
Timeline of periostin expression after MI in freshly isolated cardiac fibroblasts of BALB/c mice, measured by flow cytometry. Cells were extracted from BALB/c female mice*.* (**A–E)** Periostin cellular expression and MEFSK4 cellular expression were measured at each of the selected time points after the infarction. Gating thresholds were determined by isotype controls (dashed lines). Periostin expression is shown by itself and in conjunction with the surface marker MEFSK4. (**F–H**) Panels show post-MI timelines for cellular expression of periostin, MEFSK4 and their coupled expression, respectively. Protein expression values displayed as % of maximal expression. Data are expressed as the means ± SD (*n* = *3)* **p < 0.01,***p < 0.005, (Kruskal–Wallis’s multiple comparisons test, one-way ANOVA).

To establish a representative timeline of periostin upregulation in CFs after the MI, CFs were extracted from MI-induced wild-type BALB/c female mice at different time points post-MI (Fig. 5). Healthy mice that did not undergo the MI surgery served as references to the standard protein expression in a non-pathological cardiac tissue. Flow cytometry results depict an increase in periostin expression level in the infarcted heart (the relative part of periostin-expressing cells among the produced, living cell population), starting from two days after MI and lasting until seven days after MI (Fig. 5A1–D1), which is indicative of fibroblast activation. In line with the timeline reported from gene analysis (Fig. 1A), periostin expression began to decline after day 7 (Fig. 5E1). Furthermore, its highest expression levels were measured three to seven days after the MI with a considerable peak on day 7 (Fig. 5C1–D1), thereby reinforcing the specified timeframe of major periostin upregulation in the infarct heart (Fig. 5F). Expectedly, because post-MI, periostin is thought to be expressed specifically by activated fibroblasts^5,12,15,20,25,26^, when focusing periostin expression to cells expressing also MEFSK4, the periostin expression timeline is preserved: three days after the MI, the population of cells expressing both MEFSK4 and periostin was markedly enhanced (Fig. 5C3 compared to Fig. 5A3), continuing its growth to seven days after the MI (Fig. 5D3). On day 14, the population size was also diminished (Fig. 5E3), overall correlating with the above-mentioned timeline of periostin upregulation in the infarcted heart (Fig. 5H compared to Fig. 5F). Similar to periostin, MEFSK4 expression escalated moderately during the first three days after the MI (Fig. 5A2–C2), followed by a noticeable spike to its maximum rate on day 7 (Fig. 5D2). As MEFSK4 expression was shown to be independent of cell activation^28^, and indeed, its upregulation was not disabled by periostin upregulation (Fig. 5D1,D2), this escalation is most likely the outcome of the fibroblast proliferation that is known to take place in response to the insult and in parallel with periostin^21^. Fourteen days after the MI, MEFSK4 expression was again reduced (Fig. 5E2), marking the end of its post-MI upregulation trend (Fig. 5G). The larger MEFSK4+ population compared to that of periostin + at each of the time points can also be explained by MEFSK4 antibody recognizing murine CFs regardless of their activation status, as cells were originally harvested from the prepared whole ventricle extract (Fig. 2), and non-activated fibroblasts (negative to periostin) were also included in the analyses. The protein expression timelines that were inferred from analyses of the C57BL/6J mouse strain after MI (Fig. S3G–I) strongly resemble those described in BALB/c mice (Fig. 5F–H). Periostin continued to rise until it peaked on day 7, eventually fading by day 14 after MI, as shown in both gene and protein analyses. In clarifying periostin's period of activity post-MI, these findings may imply an appropriate intervention timeframe for fibrosis regulation therapy.

## Discussion

In this study, we propose for the first time a defined timeline for the upregulation of periostin production in the infarcted heart. Given the known role of periostin as a mediator in cell–matrix crosstalk and its association with fibroproliferative myocardial diseases, periostin has drawn widespread attention among researchers attempting to regulate cardiac remodeling after MI^17,18,30^. Although periostin manipulation technologies have been discussed in various contexts^2,19,20^, the correct timing and duration of periostin targeting have not been thoroughly studied. The elucidation of these parameters constitutes an important step in the development of a periostin-based therapy^30^, as affirmed by findings that, on the one hand, complete periostin deletion resulted in post-MI ventricular wall rupture while, on the other hand, periostin over-expression aggravated cardiac remodeling ^2,19^. This study unraveled the often overlooked time-limited nature of periostin expression by quantitatively estimating its production over the course of a specific period of time after the execution of surgical-MI performed in C57Bl/6J and BALB/c mice models.

By measuring the level of the *Postn* gene rather than that of its protein in the tissue, we were able to exclude periostin that had accumulated in the ECM^22^ and narrow the results to include only the periostin during its synthesis by cells to obtain a more accurate report. We found that periostin mRNA expression in the infarction region of C57Bl/6J mice increased significantly during days 4–7 after the MI and decreased on day 14. In light of the compatibility in the upregulation time and fold increase rate of collagen type 1 with periostin gene expression, it seems likely that the observed trend in periostin gene expression is representative of the collagen producing cells, i.e., activated CFs, among all of the different cell types. The expression of collagen type 1 and α-SMA in abundance on days 14–28, a period that coincided with periostin downregulation, shows that the myofibroblasts emerged from activated fibroblasts^5,31,32^. This finding supports the disputed claim^33^ that periostin marks activated fibroblasts^5,12,15,25,26^ rather than myofibroblasts.

The flow cytometry analyses identified ~ 35% of non-myocyte cells extracted from healthy hearts as fibroblasts. These results support the findings of previous studies indicating that fibroblasts comprise a minority in the non-myocyte cardiac cell contents^28,34^. Interestingly, however, researchers have reported that fibroblasts account for even lower percentages, approximately 15%, of the non-myocyte cardiac cell population^28^. Such differences are expected considering not only the wide variation found in myocyte frequency in the adult mouse heart (from 25 to 35% of all cells^28^), but also the ability of the MEFSK4 antibody to recognize both mature CFs and their embryonic counterparts, which are often referred to as resident mesenchymal cells. The use of additional markers would have increased the specificity for fibroblasts.

In the infarcted hearts, MEFSK4 expression on CFs was shown to be uninfluenced by their activation. The lower periostin + MEFSK4 + cell counts compared to those of periostin + cells at each specified time point may be partially due to periostin expression by valve interstitial cells (VICs). Though VICs are classed as fibroblasts, they are assumed to originate from endothelial cells that have undergone epithelial to mesenchymal transition (EMT)^15,20,35,36^, making them somewhat different from resident fibroblasts of the myocardium, which are derived from the mesenchymal lineage and recognized by MEFSK4^28,37^. The observation that periostin is already found on VICs during the early stages of healthy cardiac development^36^ may also explain its expression in the absence of the MI trigger (Fig. 5A1,A3). Moreover, the timeline of periostin protein expression found in CFs from the whole ventricle extract was virtually identical to that for the aforementioned gene expression by all cells of the corresponding infarction area. This observation confirms the role of activated fibroblasts as the predominant driver of post-MI periostin upregulation, and as such, they are a promising target for a periostin-based treatment aimed at regulating MI-induced fibrosis and remodeling. Furthermore, it validates the main location of these fibroblasts as the infarction region. Notwithstanding the foregoing, periostin expression on day 7 after the MI has been previously documented in macrophages as well^38^, as they may assume a fibroblast-like phenotype, possibly marking them as a potential target for periostin at this specific time point.

The similarity of the periostin timelines observed in the two tested mouse strains (C57Bl/6J and BALB/c) indicates an important constancy in cardiac periostin expression in these MI-induced mouse models, guaranteeing their relevance to further post-MI periostin research. The significance of that assurance is emphasized by the necessity to explore and comprehend the possible medical implications of periostin manipulation in terms of both ventricular instability (documented predominately in C57Bl/6J) and cardiac remodeling (mostly characteristic of BALB/c models)^24^. It should be noted, however, that the differences we observed between the two strains in initial periostin + cell population size predict possible variation in their individual reactions to periostin-targeted treatments, but how the effects of these differences might ultimately alter individual reactions to the treatment has yet to be clarified.

The establishment of a timeline for periostin expression at the cellular level in the infarcted heart is without precedent, as it is uninfluenced by periostin accumulation in the ECM, and therefore reflects its upregulation period most accurately. Using the simple method of flow cytometry, we obtained a well-defined timeframe for periostin manipulation after MI, and we showed that it supports the four differentiated states of the fibroblast under scar formation proposed by Fu et al.^21^. Relying on this timeframe, periostin-targeting strategies (especially by direct cell targeting^20^) can be optimally designed to enhance the effectiveness of the treatment and to minimize undesirable outcomes. The relatively limited duration of the timeline, however, no more than six days (days 2–7 after the MI), may render some of the proposed strategies irrelevant. To that end, we believe that the siRNA system is ideally suited to periostin-based gene therapy, since it may enable periostin inhibition that aligns well with the post-MI timeline for periostin expression. We therefore intend to investigate this option further in our ongoing research of periostin's role in post-MI cardiac fibrosis. As the siRNA system itself has therapeutic effects applicable in the treatment of short-term processes, it will hopefully facilitate wound healing without progressive fibrosis, thus establishing the basis for improved cardiac prognoses in the wake of MI. Similar to siRNAs, pharmacological agents are particularly suited when the intervention needs to be restricted in either time or compartment. PNDA-3, known to bind the FAS1 domain structure of periostin to interfere with its function^39^, may therefore be another preferred strategy when discussing time-limited Periostin inhibition.

### Study strengths & limitations

The results of this study are limited to the use of female mice only. The practice was restricted to females due to their greater survival rates, as males were previously shown to develop significant ventricular dilation already during the first 3 days after the MI^40,41^, and therefore increased probability of cardiac rupture^42–44^. Additionally, the variability in LAD ligation may influence both the location and intensity of the infarction, possibly affecting the expression level of fibrotic genes. The advantage of this surgical MI model, however, is its ability to mimic the clinical situation of coronary artery occlusion most appropriately. Finally, the cell extraction procedure yields all non-myocyte cell populations in the heart, making the differentiation of fibroblasts from other cell types relatively difficult. In this study we used only one marker for fibroblasts identification which is not optimal.

## Supplementary Information

Supplementary Information.

## Data Availability

All the data supporting the findings of this study are available within the article and its Supplementary Information file.

## Abbreviations

αSMA: Alpha smooth muscle actin
CFs: Cardiac fibroblasts
ECM: Extracellular matrix
GF: Growth factor
HPRT: Hypoxanthine guanine phosphoribosyl transferase
IZ: Infarct zone (of myocardium)
LCM: Laser capture microdissection
LV: Left ventricle
MI: Myocardial infarction
PCFs: Primary cardiac fibroblasts
PDGF: Platelet-derived growth factor
RZ: Remote zone (of myocardium)
TGF-β-1: Transforming growth factor, beta-1
VICs: Valve interstitial cells
