# Supplementary material for: Defining the timeline of periostin upregulation in cardiac fibrosis following acute myocardial infarction in mice
Source: Sci Rep. 2022 Dec 18;12:21863. doi: 10.1038/s41598-022-26035-y (PMC9760637; doi:10.1038/s41598-022-26035-y)
Supplement: Supplementary file 1 — Supplementary Information. [file 41598_2022_26035_MOESM1_ESM.pdf]

Supplementary Information

Defining the timeline of periostin upregulation in cardiac fibrosis following acute myocardial infarction in mice

Short Title: Post-MI periostin upregulation timeline in mice

Hadas Gil[a], Matan Goldshtein[a], Sharon Etzion[b], Sigal Elyagon[c], Uzi Hadad[d], Yoram Etzion[b,c], Smadar Cohen[a,b, d*]

[a]The Avram and Stella Goldstein-Goren Department of Biotechnology Engineering, Ben-Gurion University of the Negev, 8410501 Beer-Sheva, Israel. Emails: HG, hadasp@post.bgu.ac.il; MG, goldsmat@post.bgu.ac.il.

[b]Regenerative Medicine and Stem Cell (RMSC) Research Center, Ben-Gurion University of the Negev, 8410501 Beer-Sheva, Israel.

[c]Department of Physiology and Cell Biology, Ben-Gurion University of the Negev, 8410501 Beer-Sheva, Israel.

[d]Ilse Katz Institute for Nanoscale Science & Technology, Ben-Gurion University of the Negev, 8410501 Beer-Sheva, Israel. Email: forti@bgu.ac.il.

*Corresponding author:
Prof. Smadar Cohen, Ph.D.
E-mail: scohen@bgu.ac.il

1. Supplemental Materials and Methods

1.1. *In-vitro* study of GF-activated cardiac fibroblasts

Production of cardiac fibroblasts from healthy mice and cell culturing

10-14-week-old C57BL/6J healthy male mice (purchased from Envigo (Jerusalem, Israel)) were euthanized with isoflurane and dissected for their heart extraction. The hearts were washed in cold PBS while applying successive pressings to remove the excessive blood. Aorta and atriums were removed, and each heart was minced into pieces measuring ~2 mm and suspended in a 3-ml enzyme solution of 1 mg/ml Liberase® Thermolysin High (Sigma-Aldrich-Merck, Rehovot, Israel) and 10 μM $CaCl_2$ in HBSS. The entire contents were pipetted 12 times using a 5-ml pipette, and put in an orbital incubator shaker for 15 min (85 RPM, 37 °C). Pipetting and shaking were repeated twice more for a total shaking time of 45 min. Next, the solution was pipetted 30 more times using a 1000-μl pipette passed through a 40-μm filter and added

to 5 ml of complete fibroblasts-growth culturing medium (88% high glucose Dulbecco's Modified Eagle's Medium (DMEM) supplemented with 10% (v/v) Fetal Bovine Serum (FBS), 1% (v/v) Penicillin-Streptomycin-Neomycin (P-S) and 1% (v/v) L-glutamine 200 mM (L-Glu)). The mixture was centrifuged at 1000g for 5 min at 21 °C, the supernatant was aspirated and the cell pellet was resuspended with 10 ml of complete growth medium and seeded in a 10-ml culture plate coated with 0.1% (w/v) gelatin. 24 h after cell seeding, the medium was replaced to remove dead cells and tissue debris, after which the medium was replaced every 2-3 days. Cells were kept incubated in 5% CO_2 and ambient O_2. When they reached 70-80% confluency, cells were split using trypsin-EDTA to avoid cell contact inhibition and quiescence. PBS, HBSS and cell culturing reagents were all purchased from Biological Industries (BI, Kibbutz Beit-Haemek, Israel).

Growth Factor (GF) Activation

First- and third-passage primary cardiac fibroblasts (PCFs) were seeded to obtain a cell density of 4.5×10^4 cells/well in 0.1% (w/v) coated gelatin in 12-well-plates, after which they were left for overnight incubation to allow them to attach in a monolayer. After 20 h, the culture medium was replaced with a starvation medium, i.e., a low serum medium of 97% (v/v) DMEM supplemented with 1% (v/v) FBS, 1% (v/v) P-S and 1% (v/v) L-Glu (1 ml per well). 24 h later, the starvation medium was removed, the cells were washed once with PBS, and for the final step, the cells were then exposed to an activation medium, i.e., a low serum medium supplemented with growth factors: 10 ng/ml Mouse-TGF-β1 (BioLegend, Petach-Tiqva, Israel); 20 ng/ml Mouse-PDGF-B (BioLegend). Cells were incubated for either 24 or 48 h.

Gene Expression Analysis

RNA Extraction of cultured PCFs: Culture medium was removed from cells, which were then washed once with cold PBS. Approximately 1×105 PCFs (from growth-factor activation) were pelleted and their mRNA was extracted using cell lysis buffer, homogenization, ethanol precipitation and RNA mini-column for RNA binding and elution, according to manufacturer's instructions (PureLink™ RNA Mini Kit, Thermofisher Scientific, Kiryat Shmona, Israel). RNA concentrations were measured in NanoDrop (Thermofisher Scientific).

Reverse Transcription: To receive stable cDNA samples for real-time PCR, a reverse transcription PCR was performed on equal amounts of the RNA samples: 100 ng of each RNA sample was diluted with Diethyl Pyrocarbonate (DEPC)-treated water (RNAse free, BI) for a final volume of 10 µl, and were reverse transcribed using a High Capacity cDNA Reverse Transcription Kit (Applied Biosystems, Foster city, CA).

Quantitative Real-Time PCR (qPCR): cDNA samples were diluted in molecular biology grade water (DNase RNase-free, BI) by a 1:1.5 ratio. To determine mRNA levels, real time-PCR analysis was performed in the StepOnePlus Real-Time PCR system (Applied Biosystems) using TaqMan® probe assay reagents (TaqMan™ Fast Advanced Master Mix, Applied Biosystems) and a specific TaqMan gene expression probe (Applied Biosystems) for each tested gene as detailed in Table S1. GAPDH was used as the reference housekeeping gene (endogenous control for normalization) to calculate the ΔCt and the fold change of mRNA expression (ΔΔCt) relative to the negative control group. Each 10 µl of reaction medium contained 2 µl cDNA (5 ng).

Supplemental Table S1 Taqman gene expression assays specification

Assay ID	Gene Symbol	Gene Name	Cardiac cells expressing the gene	Species
Mm01284924_m1	Postn	Periostin; osteoblast specific factor	Activated fibroblasts[1][2][3][4][5]	Mouse
Mm00801666_g1	Col1a1	Collagen; type I; alpha 1	Activated fibroblasts[1][6]; myofibroblasts[7][8]	Mouse
Mm01178820_m1	Tgfb1	Transforming growth factor; beta 1	Activated fibroblasts[9]; myofibroblasts[9]	Mouse
Mm01546133_m1	Acta2	Actin; alpha 2; smooth muscle; aorta	Myofibroblasts[1], pericytes[1]	Mouse
Mm99999915_g1	Gapdh	Glyceraldehyde-3-phosphate dehydrogenase	All cell types (housekeeping gene)[10][11]	Mouse
Mm00446968_m1	Hprt	hypoxanthine guanine phosphoribosyl transferase	All cell types (housekeeping gene)[12]	Mouse

1.2. Experimental Myocardial Infarction

Echocardiography

LV Trace of Parasternal Long Axis B-Mode (EF>40%)

LV Trace of Parasternal Long Axis B-Mode (EF<40%)

LV Trace of Parasternal Long Axis M-mode (EF<40%)

1.3. *Ex-vivo* study of activated cardiac fibroblasts after MI

Gene Expression Analysis

Reverse transcription and quantitative real-time PCR (qPCR) were performed as described in section 1.1 above. HPRT was used as the reference housekeeping gene.

2. Supplemental Results

2.1. *In-Vitro* studies of Activated Fibroblasts

An *in-vitro* cell culture system was chosen as the first step in mimicking this described post-MI activated profile of cardiac fibroblasts and to confirm the increase in the production of periostin (and other fibrotic markers). Gene expression analysis was conducted on cultured primary cardiac fibroblasts (PCFs) isolated from healthy mice, which were then activated with growth factors present in organ fibrosis: TGF-β1, considered the most potent stimulator of myofibroblast differentiation[13][14], and PDGF-BB, known for its mitogenic effect on cells[15]. The treatment with GFs had several effects on the PCFs (Fig. S1): PCF stimulation with TGF-β1 induced an elevated expression of the fibrosis-related genes α-SMA and POSTN compared with the PDGF-BB treatment and the negative control. This was observed in both third passage cells (P.1, Fig. S1C-D) and first passage cells (P.3, Fig. S2C-D), wherein the P.3 cells exhibited higher fold increase values than the P.1 cells and reached maximum activation in shorter times (24 h post-activation in P.3 cells vs. 48 h post-activation in P.1 cells). Col1A1 mRNA expression levels were similar among all of the treatment groups, though a slight increasing trend was noted in the TGF-β1 induced cells (Fig. S1A, Fig. S2A).

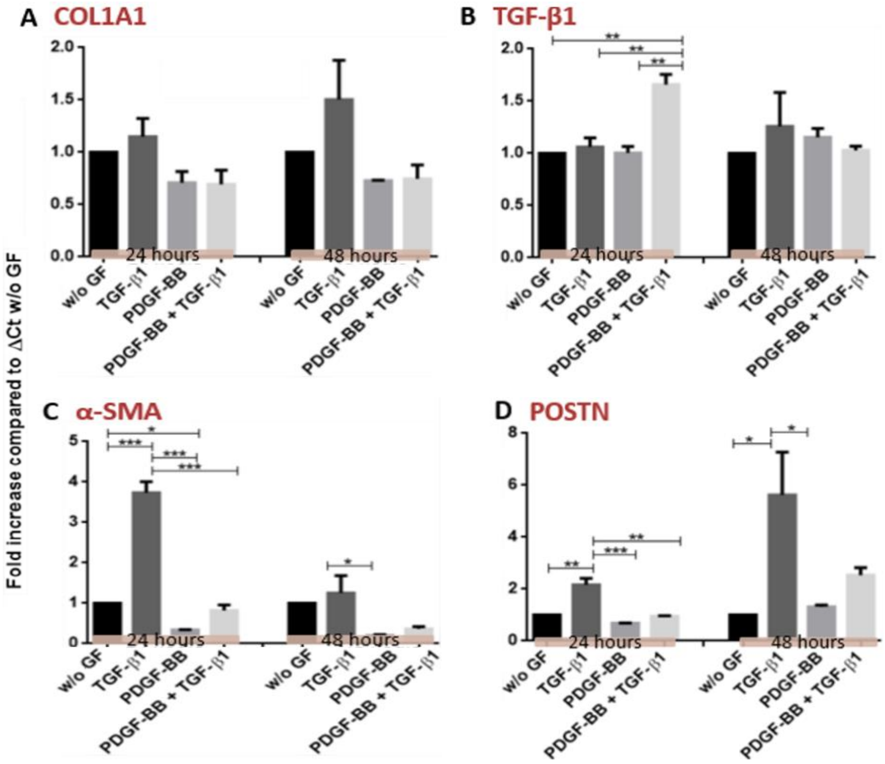

Supplemental Fig. S1 Periostin and other fibrotic gene expression after GF stimulation of third passage PCFs. PCFs from healthy adult mice (C57BL/6J males) were analyzed after 24/48 hours with GF treatment (10 ng/ml TGF-β1, 20 ng/ml PDGF-B, or both simultaneously). Data represent fold change increase of exhibited genes: **A**, COL1A1; **B,** TGF-β1; **C**, α-SMA; and **D**, POSTN, compared to group untreated with growth factors (Tukey's multiple comparisons test, means ± SD, **** = $p < 0.0001$, *** = $p < 0.001$, ** = $p < 0.01$, * = $p < 0.05$).

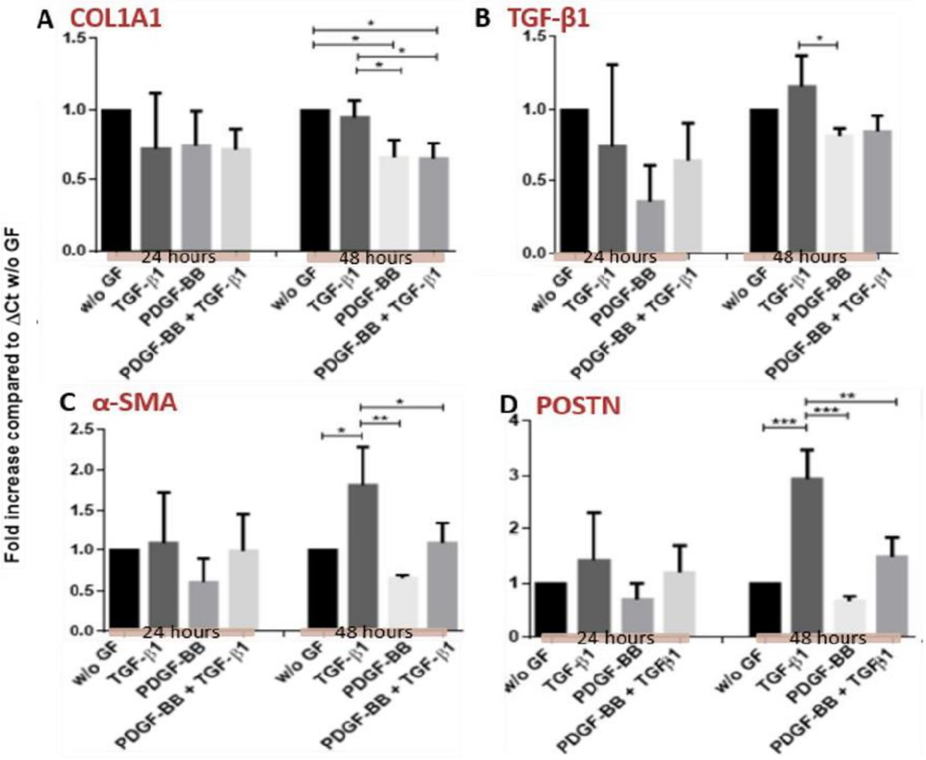

Supplemental Fig. S2 Periostin and other fibrotic gene expression after GF stimulation in first passage PCFs. PCFs from healthy adult mice (C57BL/6J males) were analyzed after 24/48 hours with GF treatment (10ng/ml TGF-β1, 20ng/ml PDGF-B, or both simultaneously). Data represent fold change increase of exhibited genes: **A**, COL1A1; **B**, TGF-β1; **C**, α-SMA; and **D**, POSTN, compared to group untreated with growth factors (Tukey's multiple comparisons test, means ± SD, **** = $p < 0.0001$, *** = $p < 0.001$, ** = $p < 0.01$, * = $p < 0.05$).

2.2. *Ex-vivo* study of activated cardiac fibroblasts after MI

Post-MI periostin expression

To establish a representative, post-MI timeline of periostin upregulation in cardiac fibroblasts, production and identification of cardiac fibroblasts was executed on MI-induced wild-type C57BL/6J female and male mice at different time points after the MI (Fig. S3). Healthy mice that did not undergo the MI surgery used as references to the standard protein expression in non-pathological cardiac tissue. Flow cytometry results depict an increase in periostin expression levels in the infarcted heart (the part of the produced living cell population that comprises periostin-expressing cells) starting on day 2 after MI and lasting up to 7 days after MI (Fig. S3A1-E1) that is indicative of fibroblast activation. In line with the timeline found via gene analysis (Fig. 1A),

periostin expression began to decline after day 7 (Fig. S3F1). Furthermore, periostin's expression levels peaked 5-7 days after the MI with a considerable peak on day 7 (Fig. S3C1-E1), hereby reinforcing the specified timeframe of periostin major upregulation in the infract heart (Fig. S3G). Expectedly, since periostin is known to be expressed after MI by activated fibroblasts[1][2][3][4][5][16], measurements of periostin expression in cells that also express MEFSK4 show that the timeline of periostin expression is preserved: five days after the MI, the population of cells expressing both MEFSK4 and periostin was markedly enhanced (Fig. S3C3 compared to Fig. S3A3), continuing its growth to seven days after the MI (Fig. S3E3). On day 14, the population size was again diminished (Fig. S3F3), overall correlating with the above-mentioned timeline of periostin upregulation in the infracted heart (Fig. S3I compared to Fig. S3G). Lower count of periostin+MEFSK4+ cells compared to the general periostin+ cell portion in each specified time point is possibly due to some extent of periostin expression contributed by valve interstitial cells (VICs). Though VICs are classed as fibroblasts, they are assumed to originate from endothelial cells that have undergone epithelial to mesenchymal transition (EMT)[3][16][17], making them somewhat different from resident fibroblasts of the myocardium which are derived from the mesenchymal linage and recognized by MEFSK4[3][17][18][19]. Since periostin is found on these cells from early stages of healthy cardiac development[20], this may also explain periostin expression in the absence of the MI trigger (Fig. S3A1, A3). Similar to periostin, MEFSK4 expression escalated during the first six days after the MI (Fig. S3A2-D2) and peaked on day 7 (Fig. S3E2). As MEFSK4 was shown to be independent of cell activation[19], and indeed its upregulation was not disabled by periostin upregulation (Fig. S3E1, E2), this escalation is most likely the outcome of fibroblasts proliferation, known to take place in response to the insult along with periostin expression[21]. Fourteen days after the MI, MEFSK4 expression declined (Fig. S3F2), marking the end of the post-MI upregulation of MEFSK4 (Fig. S3H). The larger MEFSK4+ cell populations compared to those of periostin+ at each specified time point can be explained by the recognition of murine cardiac fibroblasts by the MEFSK4 antibody, regardless of fibroblast activation status. Insofar as cells were originally extracted from whole ventricles (Fig. 2), non-activated fibroblasts (negative to periostin) were also included in the analyses. Protein expression timeframes inferred from analyses post-MI of the BALB/c mouse strain (Fig. 5F-H) show a strong resemblance to that described in C57BL/6J mice (Fig. S3G-I). The results of both gene and protein analyses – which showed that periostin expression levels

continued to rise until they peaked on day 7, later fading by day 14 after the MI – clarifies its post-MI period of activity period and imply a potentially adequate timeframe for intervention therapy.

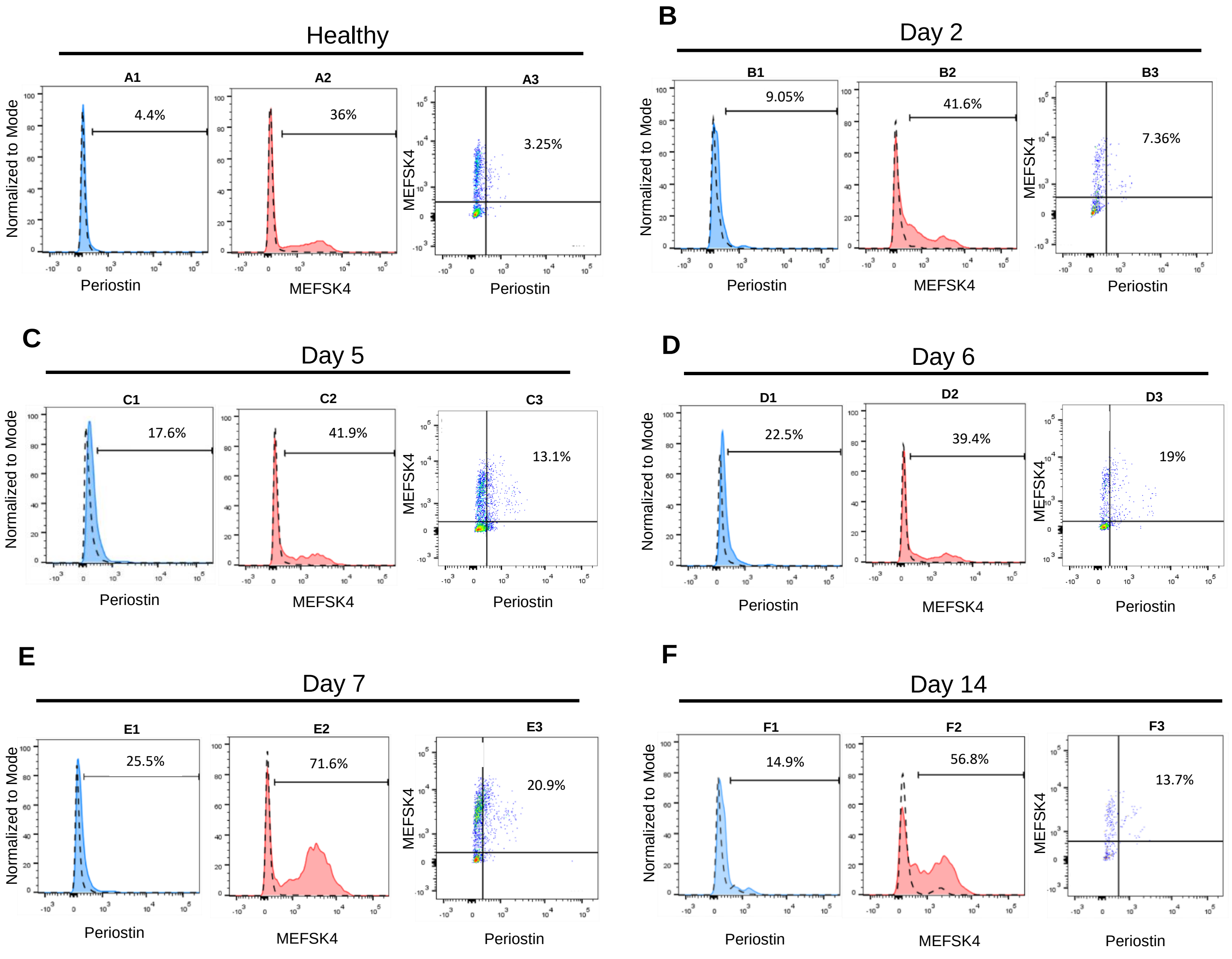

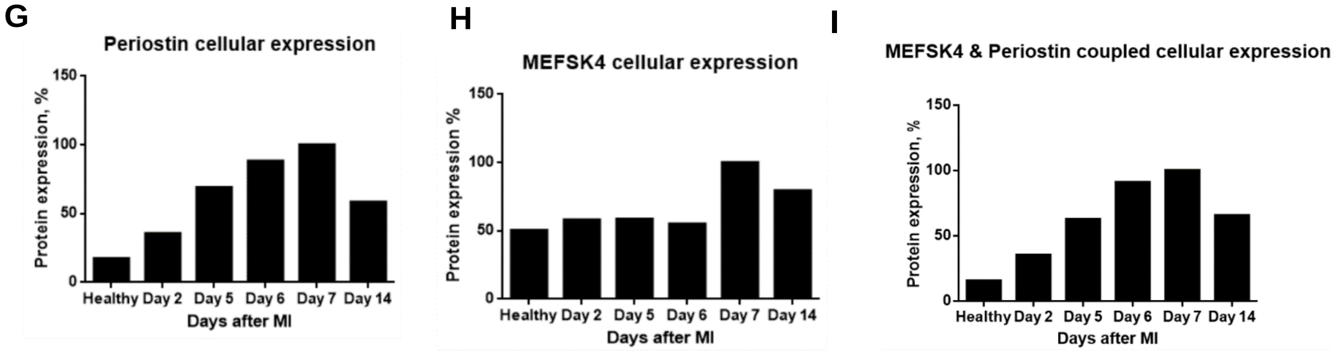

Supplemental Fig. S3 Post-MI periostin expression timeline in freshly isolated non-myocyte cardiac cells of C57BL/6J, measured by flow cytometry. Cells were extracted from C57BL/6J female mice (*n = 1*). **A-F,** Cellular expression levels of periostin and MEFSK4 were measured at each selected time point after the infarction. Gating thresholds were determined by isotype controls (dashed lines). Periostin expression by itself and in conjunction with the surface marker MEFSK4 are shown. **G-I,** Post-MI timelines for the cellular expression of periostin, MEFSK4 and their coupled expression, respectively. Protein expression is displayed as % of maximal expression.

3. Supplemental References

1. Forte, E., Furtado, M. B. & Rosenthal, N. The interstitium in cardiac repair: role of the immune–stromal cell interplay. *Nat Rev Cardiol* **15,** 601–616 (2018).
2. Conway, S. J. et al. The role of periostin in tissue remodeling across health and disease. *Cell Mol Life Sci* **71,** 1279–1288 (2014).
3. Snider, P. et al. Origin of cardiac fibroblasts and the role of periostin. *Circ Res* **105,** 934–947 (2009).
4. Doppler, S. A. et al. Cardiac fibroblasts: more than mechanical support. *J Thorac Dis* **9,** S36–S51 (2017).
5. Hortells, L. et al. A specialized population of periostin-expressing cardiac fibroblasts contributes to postnatal cardiomyocyte maturation and innervation. *PNAS* **117,** 21469–21479 (2020).
6. Ivey, M. J. & Tallquist, M. D. Defining the cardiac fibroblast. *Circ J* **80,** 2269–2276 (2016).
7. Bartold, P. M. & Raben, A. growth factor modulation of fibroblasts in simulated wound healing. *J Periodontal Res* **31,** 205–216 (1996).
8. Midwood, K. S., Valenick, L. W. & Schwarzbauer, J. E. Tissue repair and the

dynamics of the extracellular matrix. *Int J Biochem Cell Biol* **36,** 1031–1037 (2004).

9. Talman, V. & Ruskoaho, H. Cardiac fibrosis in myocardial infarction—from repair and remodeling to regeneration. *Cell Tissue Res* **365,** 563–581 (2016).
10. Li, M., Rao, M., Chen, K., Zhou, J. & Song, J. Selection of reference genes for gene expression studies in heart failure for left and right ventricles. *Gene* **620,** 30–35 (2017).
11. Brattelid, T. et al. Reference gene alternatives to Gapdh in rodent and human heart failure gene expression studies. *BMC Molecular Biol* **11,** 22 (2010).
12. Everaert, B. R., Boulet, G. A., Timmermans, J. P. & Vrints, C. J. Importance of suitable reference gene selection for quantitative real-time PCR: special reference to mouse myocardial infarction studies. *PLOS ONE* **6,** e23793 (2011).
13. Verrecchia, F. & Mauviel, A. Transforming growth factor-beta and fibrosis. *World J Gastroenterol* **13,** 3056–3062 (2007).
14. Sandbo, N. & Dulin, N. The actin cytoskeleton in myofibroblast differentiation: ultrastructure defining form and driving function. *Trans Res* **158,** 181–196 (2011).
15. Klinkhammer, B. M., Floege, J. & Boor, P. PDGF in organ fibrosis. *Mol Aspects Med* **62,** 44–62 (2018).
16. Kaur, H. et al. Targeted ablation of periostin-expressing activated fibroblasts prevents adverse cardiac remodeling in mice. *Circ Res* **118,** 1906–1917 (2016).
17. Norris, R. A., et al. Periostin promotes a fibroblastic lineage pathway in atrioventricular valve progenitor cells. *Dev Dyn* **238,** 1052–1063 (2009).
18. Schwanekamp, J. et al. TGFBI functions similar to periostin but is uniquely dispensable during cardiac injury. *PLOS ONE* **12,** e0181945 (2017).
19. Pinto, A. R. et al. Revisiting cardiac cellular composition. *Circ Res* **118,** 400–409 (2016).
20. Markwald, R. R. et al. Role of periostin in cardiac valve development. *Adv Exp Med Biol* **1132,** 177–191 (2019).
21. Fu, X. et al. Specialized fibroblast differentiated states underlie scar formation in the infarcted mouse heart. *J Clin Investig* **128,** 2127–2143 (2018).
